# Topic Modeling for Analyzing Patients’ Perceptions and Concerns of Hearing Loss on Social Q&A Sites: Incorporating Patients’ Perspective

**DOI:** 10.3390/ijerph17176209

**Published:** 2020-08-27

**Authors:** Junghwa Bahng, Chang Heon Lee

**Affiliations:** 1 Department of Audiology and Speech Language Pathology, Hallym University of Graduate Studies, HUGS Center for Hearing and Speech Research, Seoul 06157, Korea; 2 College of Business and Economics, United Arab Emirates University, Al Ain 15551, UAE

**Keywords:** hearing loss, latent topic, LDA, topic modeling, social Q&A

## Abstract

Hearing loss is the most common human sensory deficit, affecting normal communication. Recently, patients with hearing loss or at risk of hearing loss are increasingly turning to the online health community for health information and support. Information on health-related topics exchanged on the Internet is a useful resource to examine patients’ informational needs. The ability to understand the patients’ perspectives on hearing loss is critical for health professionals to develop a patient-centered intervention. In this paper, we apply Latent Dirichlet Allocation (LDA) on electronic patient-authored questions on social question-and-answer (Q&A) sites to identify patients’ perceptions, concerns, and needs on hearing loss. Our results reveal 21 topics, which are both representative and meaningful, and mostly correspond to sub-fields established in hearing science research. The latent topics are classified into five themes, which include “sudden hearing loss”, “tinnitus”, “noise-induced hearing loss”, “hearing aids”, “dizziness”, “curiosity about hearing loss”, “otitis media” and “complications of disease”. Our topic analysis of patients’ questions on the topic of hearing loss allows achieving a thorough understanding of patients’ perspectives, thereby leading to better development of the patient-centered intervention.

## 1. Introduction

Hearing loss is the most common human sensory deficit, affecting normal communication. Hearing loss is a multifactorial disorder caused by both genetic and environmental factors [1]. According to recent reports from the World Health Organization (WHO) [2], the global prevalence of hearing loss has increased rapidly over the last decade. The WHO estimated that, in 2018, 6.1% of the world’s population or 466 million people experienced some degree of hearing loss. Furthermore, it is expected that the number of people with hearing loss will rise to 900 million by 2050 [2]. Hearing loss can negatively affect the quality of life as it has adverse effects on communication performance as well as emotional and social functions [3,4]. Thus, understanding the patients’ perspective of their hearing loss is an essential step toward patient improvement.

Developing adequate healthcare and public health promotion interventions requires not only an in-depth knowledge of diseases and traits, but also a comprehensive understanding of patients’ perceptions, misconceptions, concerns, and needs about the diseases. Clinical and behavioral interventions are more effective when seeking to improve outcomes that are central to patients’ experiences and perspectives [5]. A review of prior literature showed that patient-centered interventions are responsive to patients’ needs and thus, patients are encouraged to actively participate in the research process, especially regarding the identification of salient issues and concerns. By recognizing the importance of patient engagement, scholars have increasingly paid attention to electronic patient-authored texts as sources of valuable information [6].

With the advances in Internet and communication technologies, healthcare professionals and patients can easily communicate about patient-provided data. In addition, patients not only can easily generate and share their health data and concerns with others, but also look for relevant medical information from peers who have experienced a similar diagnosis, set of symptoms or treatments. Fox and Duggan [7] investigated the use of online resources and found that 72% of Internet users used the Internet to search for health-related information, and 39% of these online health seekers looked for health information related to their own health or medical situation [7]. Research about health communication has demonstrated that health information exchanged on the Internet is a useful resource to examine patients’ perceptions, concerns, and needs. Among Internet-based platforms, community-based question-and-answer forums are increasingly becoming popular as a medium for exchanging health information [8,9].

It is not surprising that patients with hearing loss or hearing difficulties use social media channels and social Q&A, such as Yahoo! Answers in English, Baidu Knows in Chinese, and Naver Knowledge-IN in Korean, to exchange information to address their concerns about hearing problems. Prior research has demonstrated that a collection of questions that patients post on social Q&A provides not only interesting but also important information to health professionals [9,10,11]. Topic analysis of patients’ questions on the topic of hearing loss allows for achieving a thorough understanding of patients’ perspectives, thereby leading to better development of the patient-centered intervention.

In this paper, we investigate the electronic patient-authored questions on social Q&A sites by applying the natural language analytics. This study adopts a multi-component semantic and computational linguistics method to discover and analyze themes or topics from hearing loss-related health conversations in the social Q&A sphere. Computational linguistics analysis is suitable to process unstructured textual data and identify hidden patterns in the data. Notably, a probabilistic topic modeling is applied to answer our research questions—topic prevalence, topic correlation, and topic evolution: what are hearing loss-related topics being asked or communicated in the social Q&A sphere? What are the prevalent topics in the hearing loss health community? How are those topics interrelated?

First, to the best of our knowledge, no prior studies have examined questions and conversations on social media Q&A as a viable source of understanding patients’ prevalent concerns on hearing loss. By applying LDA topic modeling for text analysis techniques on electronic patient-authored hearing loss questions, our present study extracts latent topics and their dominant words, which might provide useful insights for understanding patients’ perspectives and concerns on hearing loss. Second, our results reveal that patients post about the relationship between hearing loss and other diseases such as otitis media, tinnitus, and chronic kidney disease. These topic relations between categories have implications for the development of a patient-centered intervention. Lastly, our present research demonstrates evidence that topic model analysis techniques applied to electronic patient-authored questions are effective in studying patient-focused engagement strategy. The extant literature on hearing loss indicates that survey-based methods have been a primary study design to assess patients’ concerns and perceptions about hearing loss and related issues. Using this traditional engagement strategy is costly to capture and analyze a massive volume of patients’ concerns. Data analytics on patient-authored texts on social Q&A have the potential to improve patient-centered outcome research.

## 2. Related Literature

### 2.1. Hearing Loss

Hearing loss is the most common sensory deficit. Among 432 million people with hearing loss, 93% of them are adults, and 54% of them are male—over 5% of the world’s population have hearing loss. Furthermore, it is estimated that the number of people with hearing loss will rise to over 900 million by 2050. Untreated hearing loss can negatively affect individuals’ communication performance and thus the quality of life in individuals and their families. Hearing loss can be reduced speech understanding, declined acoustic information, and impaired localization of sound sources [12]. Hearing loss is associated with comorbidities such as social isolation, loneliness [13], depression [14,15,16], balance problem [17], acoustic neuroma(vestibular schwannoma), multiple sclerosis, cardiovascular disease [18], and diabetes [19,20]. Recently, a growing body of research has shown that hearing loss and dementia are related [21,22,23,24]. Hearing loss in later life is one of the factors that play an important role in decreasing cognitive ability and developing dementia.

Hearing loss can be caused by damage to any portion of the peripheral and central auditory systems. The main causes of sensorineural hearing loss are degenerative processes associated with aging, genetic mutations, noise exposure, exposure to therapeutic drugs that have ototoxic side effects, and chronic conditions [25]. The most common cause of hearing loss is aging [17]. Age-related hearing loss, generally referred to as presbycusis, typically arises from gradual changes in the inner ear, affecting the sum of sensory, neural, and metabolic causes. Additionally, other factors such as ear diseases and the effects of noise exposure may affect people at all ages and stages in life [26]. Noise-induced hearing loss is caused by loud noise exposure for more extended periods. It has been suggested that more than 12% of the global population is at risk for hearing loss from noise [27]. The WHO estimates that one-third of all cases of hearing loss can be attributed to noise exposure.

Several options are available for hearing loss, ranging from medical treatment to listening devices such as hearing aids and cochlear implants. Treatment depends on the cause and severity of hearing loss. For age-related and noise-induced hearing loss, hearing cannot be treated, but hearing can be restored after using hearing aids or cochlear implantation [25].

### 2.2. Social Q&A Community

Social Q&A is an online question-and-answer platform enabled by Internet and Web technologies. It is a community-driven platform that allows online users to exchange information by asking questions and providing answers [28,29]. It is open to the public, where interested parties submit questions to be answered by other fellow online users around the world. Over the last decade, social Q&A has gained popularity, and the number of visits to the top Q&A sites such as Yahoo! Answers has increased dramatically [30]. For example, Yahoo! Answer includes more than 300 million questions and 90 million unique users worldwide as of 2012 since the service launched in 2005 [10]. Another popular social Q&A site launched in 2006, Wiki Answers, has 17 million answers posted. Questions and answers on topics ranging from education to diet become a source of rich experience and opinion for anyone with similar concerns or problems. Naver is the largest online platform in South Korea, referred to as the “Google of South Korea”, and Naver provides the Social Q&A platform, Knowledge-iN. Users can post any topics on Knowledge-iN, and professionals or people who know the issues make comments and provide information and solutions, in content-centered platforms, and then users select the most valuable answers, and respondents earn awards or points.

### 2.3. Social Q&A Log Analysis

According to the Pew Internet and American Life Project data, more than 70% of Internet users use the Internet to search for health or medical information [7]. Increased use of such online platforms such as social Q&A sites leads to the generation of unprecedented volumes of information about symptoms, treatments, and health directly from patients, which is generally referred to as electronic patient-authored text [6,31]. As the volume of potentially valuable patient-authored text on social Q&A is growing, more researchers have paid attention to identifying the potential of online data sharing platforms for education and health service. Online patient narratives are a reliable data source for detecting disease trends and identifying medical terms [31]. Moreover, novel insights into patients’ treatment decisions and drug-treatment effects were discovered on PatientsLikeMe [32].

A review of the literature on the electronic patient-authored text on the social Q&A community indicates that the existing research streams can be divided into content-centered (e.g., question and answer narratives) and user-centered (e.g., questioners, answerer, and the community) studies. The content-centered studies have mainly focused on three areas: (1) detection of diverse types of health-related questions and answers [9,33,34]; (2) identification of medical concepts in the patient-authored text [31]; and (3) evaluation of the quality of questions and answers with a distinct set of criteria [35,36].

The first type of research has examined electronic patient-authored questions and answers from social Q&A sites to detect health-related hot topics [33,34,37]. Lu et al. (2013) applied text clustering techniques to detect disease topics such as lung cancer, breast cancer, and diabetes, and related symptoms, medical tests, drugs, procedures, and complications. Sadah et al. [33] identified a set of popular topics and associated sentiments based on the patients’ demographics. The second type has focused on identifying medically relevant terms and mapping words from the patient-authored text to medical concepts [9,31]. A language gap between patients and health care professionals is known to hinder effective communication between the two groups, so identifying and bridging the vocabulary gap is crucial [31]. Park et al. [8] applied the named entity recognition method to identify medical terms in their collected diabetes dataset and then map the identified terms to the formal medical vocabularies in the Unified Medical Language Systems (UMLS). Lastly, with concerns about the quality of both health-related questions and answers, researchers have proposed a diverse set of quality criteria and empirically examined them [36,38]. Harper et al. [37] employed supervised machine learning algorithms to distinguish information and conversational intent questions automatically. Their findings show evidence that conversational questions yield a lower archival value than informational questions.

## 3. Methods

**Figure 1:**
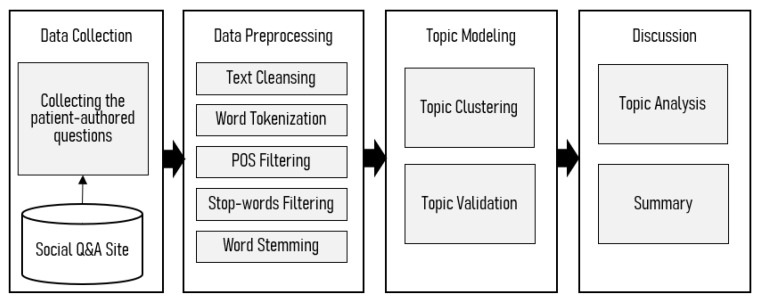
Overall research flow.

Our approach carries our semantic and linguistic analysis to reveal the health characteristics of patients’ questions in online textual questions containing hearing loss-related words. The present study consists of three phases: data collection, topic discovery, and topic extraction. Figure 1 shows the overall procedure of the research analysis.

### 3.1. Data Collection

Electronic patient-authored texts on the topic of hearing loss were collected from the social Q&A, namely Naver Knowledge-iN. Launched in 2002, Naver Knowledge-iN is the largest social Q&A community platform in South Korea, where online users can post and share questions related to various topics ranging from insurance policy to medical treatment. Health topics are popular among the questioners. To collect research data, we developed a software program to access and gather the questions posted from 2009 to 2019 on Naver Knowledge-iN. We collected 68,327 questions using the key word of “hearing loss”. Repeated or duplicated questions posted were excluded. In addition, questions were excluded if a question contained less than 10 words. As a result, our final sample dataset consists of 65,842 questions that were analyzed for this study.

### 3.2. Topic Discovery via Latent Dirichlet Allocation (LDA)

To discover the topics from the collected textual questions, we utilized a topic modeling approach that clusters the semantically associated words with “hearing loss” into subtopics. Topic modeling has been widely applied in health and medical domains such as extracting relevant clinical concepts from patient health records [31], discovering health topics in social media [11,39,40] identifying emerging patterns of clinical events [41], and detecting new disease breakout [42]. Among diverse topic modeling techniques, Latent Dirichlet Allocation (LDA) [43] has gained popularity as a tool for automatic text summarization and visualization. In this study, we apply the LDA model to extract topics from the collected corpus.

The automatic text analysis method is usually divided into supervised and unsupervised methods. The unsupervised method does not classify the text content in advance but reduces the dimension of the text through statistical probability inference and explains the text as a whole by means of the reduced dimension theme. The LDA model is an unsupervised machine learning method that uses a bag-of-words representation method. It utilized a latent variable *topic* between observed variables *document* and *word* to explain the semantic topic distribution of documents. The LDA modeling approach considers each document to be presented as a random mixture over latent topics, where each topic is characterized by a probability distribution over words.

**Figure 2:**
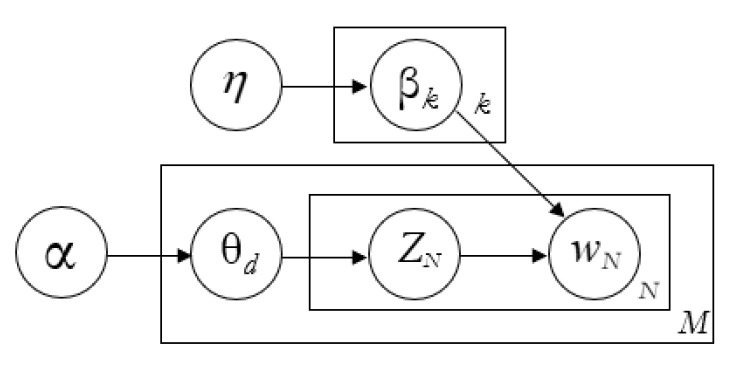
Plate model representation of Latent Dirichlet Allocation (LDA).

LDA is a generative probabilistic model that assigns sets of words collected from documents to be explained by unobserved topic groups that explain why some parts of the data are similar. Each document consists of a small number of different topics, and each word’s generation is attributional to one of the topics of the document. The plate diagram of the LDA model is shown in Figure 2, which helps to explain the components of the LDA model.

LDA assumes that documents and the words within them are derived from a generative probabilistic model [43]. Here, k is the number of topics. M is the number of documents and *N* is the number of words within the document. Given a corpus D consisting of M documents, with documents having $N_{d}$ having (d∈1,…, M), LDA models D according the followings.

- The number of words *N* is represented by the vector w as a *bag-of-words*.
- The model parameter $\theta_{d}$ represents topic proportions for the documents, which is a random variable drawn from a Dirichlet (α) prior with parameter α
- For each topic, the model parameter $\beta_{k}$ is the multinomial distribution of words and is drawn from the prior Dirichlet distribution using the parameter η.
- For each word in the document, the topic of the word z_ij_ is a random variable drawn from a multinomial ($\theta_{d}$) distribution. Thus, z_ij_ is the topic that generates w_ij_ having the *j* th word in the *i* th document.

(1) $$P(\theta ,\;z|\;w,\alpha ,\beta )=\frac{P\left(\theta ,\;z,w|\alpha ,\beta \right)}{P(w|\;\alpha ,\beta )}$$

### 3.3. Topic Extraction

The topic extraction is the final step of understanding and understanding the topics found. The meaning of the topic is determined by analyzing the most likely terms along with the most likely related documents. First, a thorough investigation was performed on the relevant documents to verify that the initial interpretation based on word probability was meaningful. Second, the two researchers independently interpret and label 21 topics, and each topic is classified into one of five theme categories, except for minor word differences.

## 4. Results

The LDA topic extraction results concerning the first question reveal similar dimensions to those addressed in the literature on hearing loss. The twenty-one topics resulting from the LDA computation are shown in Table 1. The lists of words that make up a particular topic are displayed below the topic title for each theme. The finding suggests that the identified themes not only resembled the categorization of hearing loss diagnosis and treatments but also cover issues on topics relating to hearing loss.

We then examined the extracted 21 topics and merged similar topics. Finally, the topics were classified into five themes, which were “sudden hearing loss”, “tinnitus”, “noise-induced hearing loss”, “hearing aids”, “dizziness”, “curiosity about hearing loss”, “otitis media” and “complications of disease”. Topics and words with probabilities for each topic are shown in Table 1.

**Theme 1. Sudden Hearing Loss.** *Four of the identified topics, namely Topics 3, 13, 17, and 22, pertain to sudden sensorineural hearing loss, commonly known as “sudden deafness”, so the main theme derived from the four topics is labelled as sudden hearing loss. Specifically, this theme addresses causes, diagnosis, medication treatments, prescriptions, and effects associated with both sudden hearing loss.*

Topic 3 contains the words related to the medication treatments of sudden sensorineural hearing loss: “steroid”, “injection”, “treatment”, “drug”, “prescription”, “shot”, and “sudden deafness”. We found that, based on the selected keywords, the main concern is related to steroid use for the treatment of sudden deafness. Steroids taken orally or given by injection are commonly used to treat patients who experience sudden deafness. Topic 3 shows evidence that people with sudden hearing loss post questions to learn more about the effectiveness of treatment with steroid drugs and direct injections into their ears.

Topic 13 reflects diagnosis and test related to sudden hearing loss with keywords specifically “diagnosis”, “cause”, “hospital”, “test”, “result”, “nose”, and “infection”. It is evident that patients who have been diagnosed with sudden deafness recently posted questions about what causes sudden deafness.

Topic 17 and 21 represent prescriptions and side effects, respectively. Terms such as “side effect”, “treatment”, “prescription”, “drug”, “worry”, and “recovery” explain that patients with sudden deafness express concerns about the side effects of steroid and hearing recovery. A greater portion of patients diagnosed with sudden deafness have expressed concerns about the side effects of steroid use and have posted questions about whether their steroid treatment is appropriate in terms of frequency and dosage.

**Theme 2. Tinnitus.** *Four of the identified topics, namely Topics 1, 6, 15, and 19, pertain to tinnitus, so the central theme derived from the four topics is labeled as tinnitus. Specifically, the tinnitus theme addresses symptoms, causes, and treatments associated with both tinnitus and hearing loss.*

Topic 1 and 6 represent issues of hearing loss, with tinnitus symptoms and problems due to tinnitus such as “ringing”, “buzzing”, “sound”, “loud”, “noise”, “sleep”, “hissing”, “night”, “severe”, and “stress”. Interestingly, the keywords identified in the two topics show that questioners or patients in social Q&A detail their hearing problems with specific vibrations and sounds (e.g., “ringing”, “buzzing” and “hissing”), and specify a level of severity when posting a tinnitus symptom. Tinnitus, commonly referred to as buzzing in the ears, is “often accompanied by hearing loss but not everyone with hearing loss experiences tinnitus” [44]. However, the results indicate that a significantly greater portion of patients who experience tinnitus are involved in the hearing loss topic. Additionally, patients seem to be more stressed at night due to tinnitus and worry that severe or persistent tinnitus is a sign of going deaf.

Topic 15 shows the keywords involved in treatments of tinnitus. This included words such as “treatment”, “hospital”, “doctor”, “food”, “audiologist”, “pain”, and “doctor”. The topic results show that patients are in search of diverse tinnitus treatments, and specifically seek for good hospitals or doctors, and alternative treatments for tinnitus. We also found that, based on the selected keywords, effective food is a frequently occurring word, implying that tinnitus patients also search for foods to help reduce tinnitus.

Topic 19 represents a cause of tinnitus with keywords specifically including “infection”, “drug”, “earwax”, “noise”, “stress”, and “medication”. The topic results show that people want to understand what causes tinnitus. Patients who are suffering from tinnitus want to know more about whether their recent illness, such as ear infection, ear wax, or stress, causes tinnitus. Additionally, patients with tinnitus post inquiries about the relationship between tinnitus and hearing loss, and particularly ask whether tinnitus can lead to hearing loss. Most importantly, the results show that many patients with hearing loss post questions whether exposure to loud noise causes tinnitus.

**Theme 3. Noise-Induced Hearing Loss.** *Topic 2 contains terms such as “loud”, “music”, “earphone”, “headset”, “cause”, “damage”, referred to as “noise-induced hearing loss”. These words imply that users want to ask about the relationship between listening to loud music through earphones or headsets with a personal listening device and hearing loss. In addition, people want to know how long listening to music is safe for not getting hearing loss per day.*

The authors can observe the terms “shooting”, “military”, “gun”, “explosion”, “compensation”, “degree”, and “diagnosis”, “hospital” in Topic 18. These terms imply that people who are exposed to excessive and continuous noise wonder and get the information whether and how they can get any compensation with their degree of hearing loss. Notably, South Korea has adopted a conscription system. Noise-induced hearing loss is a severe disease in the military because military personnel remain in a noisy environment for the completion of their missions. Thus, the prevalence of hearing loss and tinnitus in the military is higher than the general public [45].

**Theme 4. Hearing Aids.** *Topics 8, 11, and 16 were identified as hearing aids. Hearing aids are the most popular option of treating hearing loss for improving speech understanding. Topic 8 contains the words, “hearing aids”, “cochlear implant”, “handicap degree”, “disabled”, and “support”. In South Korea, the registered disabled person with hearing loss gets the subsidy of their hearing aid expense every five years. People with hearing loss or their family members want to know whether their hearing loss degree gets financial support.*

In Topic 11, the authors also observe the term “cochlear implant” and “surgery”. In a person with severe to profound hearing loss, hearing aids do not give many benefits. So, instead of hearing aids, cochlear implantation can help to improve speech understanding to people with severe to profound hearing loss. People want to ask about alternative interventions, such as cochlear implantation surgery, other than hearing aids.

Topic 16 consists of the keywords: “hearing aids”, “recommend”, “brand”, “hospital”, and “price”. Those keywords indicate that patients post questions by asking for a specific recommendation for an excellent hearing aid with a reasonable price in social Q&A.

**Theme 5. Otitis media.** *Topics 4 and 10 include the terms related to hearing loss with symptoms of otitis media, such as “pain”, “sore throat”, “infection”, “deafen”, “headache” and “eardrum”. Otitis media is an infection of the middle ear, and it is one of the most common disease in children [46]. The symptoms of otitis media are sore throat, night restlessness, fever, and ear pain [47].*

Topic 10 also contains keywords related to the treatment of otitis media, “hospital”, “surgery”, “medication”, “test”, “after”. The results indicate that people asked about hearing loss after taking medication or undergoing surgery for otitis media. The term “hospital”, “after”, and “doctor” imply that, right after visiting doctors, users ask questions about otitis media treatment, such as drugs and surgery. Mild hearing loss can come with otitis media, and usually, hearing loss recovers after otitis media is cured. However, if otitis media occurs repeatedly, it causes permanent hearing loss [48].

**Theme 6. Dizziness.** *Topic 9 contains the words “dizzy”, “cause”, “headache”, “awake”, “morning”, “severe” and “sudden” referred to as “dizziness”. Topic 14 also contains the keywords “headache”, “stress”, and “dizziness” referred to as “dizziness”. One of the most common cause of dizziness is problems of the inner ear. The vestibular system, which is responsible for balance, is located within the inner ear with the auditory system. There are many diseases that affect dizziness and hearing loss, including severe cold, and bacterial or virus infections of the inner ear. In particular, Ménière’s disease is a disorder of the inner ear that leads to dizziness and sudden hearing loss. The affected ear may lead to progressive and/or permanent hearing loss [49]. Other symptoms of Ménière’s disease are tinnitus, migraines, and nausea. Ménière’s disease is very hard to diagnose due to the fact that not all of the symptoms are shown at the early stage [50]. These words relating to topic 9 may represent the symptoms of Ménière’s disease.*

**Theme 7. Curiosity or general inquiry about hearing loss.** *Topics 5 and 20 contain a higher number of words relating to hearing loss itself. The authors could observe that users want to know about assessment results, such as the units (decibel) which are used for describing hearing loss. Additionally, the terms such as “stress”, “nerve”, “headache”, “worry” imply that users ask about their symptoms relating to hearing loss.*

The terms “treatment”, “hospital”, “communication” “learning” imply that people went to the hospital to have assessments of their hearing, but they did not fully understand their hearing loss and how to treat their hearing loss. In social Q&A, they want to get the information about their hearing loss.

**Theme 8: Complications of disease.** *Topic 7 and 12 represent the terms “chronic”, “anemia”, “cancer”, “thyroid”, “hepatitis”, “kidney” and “disease”. All the diseases in topic 12 are related to hearing loss. Hearing loss is a side effect of some chemotherapy drugs [51,52,53,54]. Additionally, anemia, iron deficiency, and thyroid hormone deficiency lead to hearing loss [55,56,57,58]. The hepatitis virus B and C are very strong risk factors of sudden sensorineural hearing loss [59]. Hearing loss is linked to chronic kidney disease [60], too. In social Q&A, patients or their family members seek the information about different complications leading to a hearing loss.*

## 5. Discussion

Hearing loss subtopics indicated that users posted about the relationship between hearing loss and hearing loss-related disease. The themes of sudden deafness, noise-induced hearing loss, and otitis media, and related complications of diseases are covered by hearing loss-related disease. Sudden deafness and otitis media themes show that users searched for social Q&A about the cause of diseases, treatment options, and side effects of the medicine. These topics confirmed that people are not well-informed about their medications. Notably, sudden deafness is considered an otologic emergency. Patients with sudden deafness can recover from the hearing loss problem if they receive appropriate treatments promptly. Prior studies reported that treatment within seven days of the onset of sudden deafness is effective for better hearing recovery [61]. The standard gold treatment of sudden deafness is oral high-dose corticosteroids [62]. Our findings reveal that users want to get the information and are worried about the side effect of the steroids, which means that patients did not get the information about their treatment and medicine.

In the theme complications, we found several keywords are related to diseases, including diabetes, anemia, cancer, thyroid, hepatitis, and kidney disease. The medications for these diseases have side effects that can cause hearing loss. Based on our findings, health providers, such as physicians, did not give enough information about treatment and medicine. Lee et al. [63] indicated that the average real consultation time for outpatients was 4.2 min in Korea, and patient satisfaction was too low regarding consultations. This consultation time is not long enough to ask questions about their treatments. The physicians need to ensure that they have enough consultation time for effective communication.

On the other hand, noise-induced hearing loss is a slow-occurring hearing loss. In subtopics of noise-induced hearing loss, users post about the causes of hearing loss, such as loud noise exposure and the usage of listening devices with headphones. People wonder how much loud noise exposure causes hearing loss. Exposure to noise increases the risk of tinnitus, as well. Noise-induced hearing loss is the only preventable loss, so education on the usage of a safe listening device is necessary [64]. For preventing noise-induced hearing loss, people are advised to avoid listening to loud music or noise above 85 dB (A) for no longer than 1 h. People need to avoid listening to music because it increases the volume when they listen to it under noisy circumstances. Interestingly, in these subtopics, we found keywords related to the military. Military personnel exposed to significant impulse noise. For this reason, in social Q&A, many people seek information about the relationship between hearing loss and military noise. The hearing conservation program needs to be developed adequately to ensure the health of military personnel in Korea.

Next, users posted the relationship between hearing loss and symptoms of hearing loss, tinnitus, and dizziness. The highest subtopics are related to tinnitus. Tinnitus is a symptom associated with ear disease, including hearing loss, but tinnitus does not cause hearing loss. More than 50 million people, with an estimated prevalence of 10–15% in adults, reported that they experienced tinnitus in the U.S. [65]. The keywords related to hearing loss indicated that people want to know about the cause of tinnitus, medicines, and difficulties caused by tinnitus. The findings show that people want to find a way to cure tinnitus. However, the treatment of tinnitus focuses on minimizing the impact and burden of tinnitus rather than the “cure” of tinnitus [65]. There are several rehabilitation methods for relieving tinnitus, such as counseling and sound therapy. Additionally, there are applications that patients can use to alleviate their tinnitus. Health providers need to inform the options of tinnitus relief.

Tinnitus is a symptom that people feel chronically, while dizziness is a symptom that people feel acutely. Our results reveal several keywords related to the symptoms of Ménière’s disease. Ménière’s disease is a disorder of the inner ear that leads to dizziness and sudden hearing loss. The affected ear may lead to progressive or permanent hearing loss [49]. Other distinct symptoms of Ménière’s disease include tinnitus, migraines, and nausea. Ménière’s disease is hard to diagnose because not all of the signs are shown at the early stage [50]. The patients who feel dizziness need to visit the Ear, Nose and Throat (ENT) doctor and check their conditions.

Lastly, people want to search for information about the treatment options of hearing loss, such as hearing aids and cochlear implants. Hearing aid usage is the most common treatment to reduce the adverse effects, including communication difficulties and cognitive decline, caused by hearing loss [66]. In addition, the use of hearing aids improves productive time use, quality of life, economic circumstances and mental health for listeners with hearing loss [67]. In our results, people searched for information on hearing aid benefits or the specification of hearing aids. Additionally, they searched about brands of hearing aids and reasonable prices for hearing aids. They also asked for recommendations regarding good hearing aid centers. Since the national insurance subsidizes all or part of the cost of purchasing hearing aids or cochlear implants for hearing impairments in Korea, users search the information about disability degree and national insurance benefit. This information should be provided to websites so that those who want to use hearing aids can accurately check the price and specifications of hearing aids and get national insurance benefits.

## 6. Conclusions

Hearing loss is the most frequently occurring human sensory deficit and has many different causes. Hearing loss is also known to be related to many other health issues. Analyzing patient-authored questions can be a useful approach to better understand patients’ perceptions, concerns and needs. Traditional surveys are limited to small sample sizes. However, social Q&A offers a new environment for patients to easily share various opinions and medical experiences, so a large volume of patient-authored data can accumulate. Exploring how patients with hearing loss use social questions and answers to find health information not only helps to identify a set of critical topics and issues for various types of research but also improves communication between patients and healthcare professionals. To the best of our knowledge, this is the first analysis of patient-authored contents on the topic of hearing loss from the social Q&A community, and these results provide valuable methodological and content insights.

This study provides a computational linguistic approach to perform an in-depth analysis using patient-authored data from sizeable online data sets. Our framework decodes public health views from hearing loss-related questions, which can be useful for developing adequate healthcare and public health promotion interventions. Our results reveal that those characterized topics ranging from sudden deafness to hearing aids are both representative and meaningful, and mostly correspond to sub-fields established in hearing science research.

## Figures and Tables

**Table 1:** The most probable keywords in the topic of LDA with 21 topics.

Topic No.	Topic Label	Keywords	Theme Category
1	Tinnitus Symptoms	ringing, buzzing, tinnitus, sound, hear, constant, continue, hissing, severe, ear	Tinnitus
2	Loud Music	loud, music, earphone, listen, eardrum, affect, damage, cause, headset, longtime	Noise-induced Hearing Loss
3	Steroids for Sudden Deafness	steroid, injection, treatment, drug, pill, prescription, shot, sudden, deafness, effect	Sudden Hearing Loss
4	Symptoms of Otitis Media	symptoms, otitis media, pain, headache, sore throat, infection, deafen, today, morning,	Otitis Media
5	Hearing Test Results	diagnosis, treatment, cure, curious, communication, hospital, learning, disability, quality, doubt	General Inquiry
6	Tinnitus Symptoms	tinnitus, loud, noise, hear, sleep, night, worse, stress, ear, feel, tired	Tinnitus
7	Complications	complication, cause, chronic, diabetes, anemia, cancer, thyroid, hepatitis, kidney, disease	Complications
8	Degree of Disability	handicap, disability, degree, disabled, support, experience, hearing, aids, cochlear, implant	Hearing Aids
9	Dizziness	dizzy, cause, awake, morning, severe, pain, balance, nausea, sudden	Dizziness
10	Otitis Media Treatments	surgery, hospital, medication, otitis media, medicine, doctor, condition, after, hearing, test	Otitis Media
11	Cochlear Implant	cochlear, implant, surgery, cost, normal, restore, wear, test, support, wonder	Hearing Aids
12	Health Screening	health, screening, increase, urine, test, radiation, surgery, complication, infection, hospital,	Complications
13	Diagnosis of Sudden Deafness	sudden, loss, diagnosis, cause, hospital, test, results, nose, ear, infection, damage	Sudden Hearing Loss
14	Headache	headache, stress, frequent, dizziness, symptom, discomfort, ménière, disease, nerve, brain	Dizziness
15	Tinnitus Treatments	tinnitus, treatment, hospital, recommend, food, symptoms, effects, audiologist, pain, doctor	Tinnitus
16	Hearing Aids Recommendation	Hearing, aids, recommend, brand, place, good, hospital, uncomfortable, price, impaired	Hearing aids
17	Treatment and Side Effects	treatment, sudden deafness, medicine, right, ear, left, test, normal, restore	Sudden Hearing Loss
18	Shooting	shooting, military, gun, explosion, damage, hospital, degree, diagnosis, protection, accident	Noise-induced Hearing Loss
19	Causes of Tinnitus	tinnitus, infection, drug, earwax, loud, noise, stress, medication, cause, sick	Tinnitus
20	General Inquiry	stress, headache, serious, nerve, exercise, job, worry, cause, diet, decibel	General Inquiry
21	Treatment and Side Effects	side effect, steroid, drug, virus, hours, memory, worry, recovery	Sudden Hearing Loss
